# The role of exon shuffling in shaping protein-protein interaction networks

**DOI:** 10.1186/1471-2164-11-S5-S11

**Published:** 2010-12-22

**Authors:** Douglas V Cancherini, Gustavo S França, Sandro J de Souza

**Affiliations:** 1 Ludwig Institute for Cancer Research, São Paulo Branch, Brazil; 2 Ph. D. Program, Department of Biochemistry, Institute of Chemistry, University of São Paulo, Brazil

## Abstract

**Background:**

Physical protein-protein interaction (PPI) is a critical phenomenon for the function of most proteins in living organisms and a significant fraction of PPIs are the result of domain-domain interactions. Exon shuffling, intron-mediated recombination of exons from existing genes, is known to have been a major mechanism of domain shuffling in metazoans. Thus, we hypothesized that exon shuffling could have a significant influence in shaping the topology of PPI networks.

**Results:**

We tested our hypothesis by compiling exon shuffling and PPI data from six eukaryotic species: *Homo sapiens*, *Mus musculus*, *Drosophila melanogaster*, *Caenorhabditis elegans*, *Cryptococcus neoformans* and *Arabidopsis thaliana*. For all four metazoan species, genes enriched in exon shuffling events presented on average higher vertex degree (number of interacting partners) in PPI networks. Furthermore, we verified that a set of protein domains that are simultaneously promiscuous (known to interact to multiple types of other domains), self-interacting (able to interact with another copy of themselves) and abundant in the genomes presents a stronger signal for exon shuffling.

**Conclusions:**

Exon shuffling appears to have been a recurrent mechanism for the emergence of new PPIs along metazoan evolution. In metazoan genomes, exon shuffling also promoted the expansion of some protein domains. We speculate that their promiscuous and self-interacting properties may have been decisive for that expansion.

## Background

In 1978, Walter Gilbert speculated that the presence of introns in eukaryotic genes would lead to non-homologous recombinations and the creation of new exon combinations [1]. Gilbert and others [1-3] reasoned that exons could code for protein functional units, and proposed that exon shuffling could be an important mechanism for the evolution of genes in which these functional units are reused in new molecular contexts. Since then, abundant evidence has accumulated showing that exon shuffling has effectively occurred along evolution, playing a crucial role in the origin of numerous metazoan proteins, among which predominate extracellular matrix, immune system and membrane receptors [4-10]. It has also become clear that exon shuffling is an important mechanism of protein domain shuffling [6,8,10], in particular for some domain types that have expanded significantly in metazoans.

Physical protein-protein interaction (PPI) is a critical phenomenon for the function of most proteins in living organisms, and the recent accumulation of PPI data from numerous small-scale and a few recent large-scale experiments now allow us to build proteome-wide PPI networks [11-15]. They help us to understand more globally the multiple roles that a protein may play within a cell and the complex interdependence that functions of several of them may bear among themselves. In the simplest and more usual form, these networks represent proteins by vertices, and a physical interaction between two proteins by an edge between the two corresponding vertices. A fundamental property of each vertex is the number of edges, a feature known as vertex degree. Numerous recent works have analyzed the major topological properties of PPI networks, and a recurring theme has been the special properties of vertices with high degree. They are called hubs and tend to be large multi-domain essential proteins [16-18]. Other investigations have shown that many domain-domain interactions are ubiquitous in PPI networks [19-21], suggesting that the reuse of these interactions has been relevant for the evolution of PPI networks of extant species.

This last relationship, together with the role of exon shuffling as an evolutionary process to make possible the reuse of domains, led us to the natural hypothesis that exon shuffling may have contributed significantly to the evolution of PPI networks. This present work is an effort to verify this hypothesis in several eukaryotic species and to discover what concrete and specific type of influence exon shuffling has had on PPI networks.

## Results and discussion

### Exon shuffling and network degree

With the purpose of generating PPI networks representative of the state-of-the-art knowledge, we compiled data from several public databases (a detailed description is available in the Methods section). Because of the incompleteness of data comparing interaction properties of several protein products of a gene we merged all data concerning proteins from a gene in just one vertex of the network, a strategy that has been adopted by others [22]. Given that we intended to focus on direct physical interactions between proteins, we excluded from our final network all mass spectrometry data, as also done by others [22,23], since this technique does not discriminate between direct and indirect physical interactions.

We included the five species that we found to be more balanced concerning a reliable PPI network and a considerably sized intron density: human (*Homo sapiens*), mouse (*Mus musculus*), the fruit fly *Drosophila melanogaster*, the nematode worm *Caenorhabditis elegans*, and the flowering plant *Arabidopsis thaliana*. We added to the analysis a sixth case: a predicted PPI network that was obtained by projecting the binary interactions of the exhaustively studied yeast *Saccharomyces cerevisiae* (an extremely intron-poor species) PPI network into the nearest ortholog genes in *Cryptococcus neoformans*, a fungal species where exon shuffling analysis was possible because of less extensive intron loss [24]. In several moments, however, the present work emphasizes the results obtained with the human PPI network, due to its higher completeness level, and to the abundance and conservation of human introns, which make exon shuffling identification more reliable.

In order to select a subset of genes enriched in exon shuffling events, we sought for the presence of homologous regions flanked by introns in coding sequences derived from non-homologous genes, as has been done by us and others [25,26]. For the sake of simplicity, we just considered one of the coding sequences of maximum length for each gene. Taking into account the pervasive prevalence of intron loss [27] and the antiquity of some exon shuffling cases [28], we did not require bilateral flanking of both homologous regions, and the number of required introns was smaller in species for which a more extensive intron loss process was known to have taken place in their evolutionary histories. Although arbitrary, this less stringent requirement is convenient to include as many probable exon shuffling cases as possible in the exon shuffling gene sets and, at the same time, to deplete the complementary gene sets in these cases.

**Figure 1:**
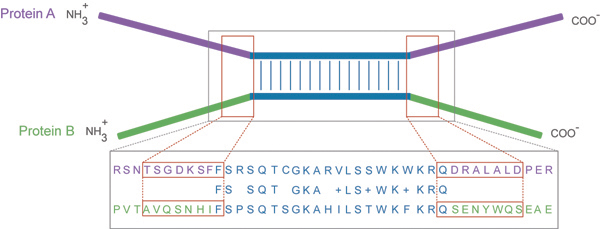
**Illustration of the method used to search for genes with evidence of exon shuffling** Local alignments were performed between proteins A and B using a set of criteria to exclude the identification of paralogs (see Methods). Homologous regions were identified (aligned amino-acid regions are shown in blue). Four flanking windows were considered in nucleotide sequences for A and B, each one corresponding to one border of the protein alignment, and surrounding the projection of this border on the nucleotide sequences. Windows around the 5’ projected border extended from 20 nucleotides in the 5’ direction to 3 nucleotides in the 3’ direction, whereas windows around the 3’ projected border extended from 3 nucleotides in the 5’ direction to 20 nucleotides in the 3’ direction. The approximate reciprocal projections of these nucleotide windows on the protein sequences are highlighted in red. If the number of windows containing introns were at least three (for human and mouse) or at least two (for the remaining species), the genes coding for both proteins were considered as ES genes. If a protein had alignments to one or several non-homologous proteins but failed to meet the above mentioned minimum intron presence criterion in all these alignments, its gene was classified as an SS gene. If a protein had no alignments to non- homologous proteins, its gene was included in the WS category.

Our strategy used local alignments to find such homologous regions resulting in a classification of protein-coding genes into three shuffling profile categories. Genes without any homologous regions were classified as without-shuffling (WS) genes. Homologous regions were then analyzed in regard to the presence of introns in four windows adjacent to their extremities (see Figure 1 for a schematic view of the strategy). Genes with at least one homologous region with a minimum number of windows containing introns were classified as exon shuffling (ES) genes. This minimum number was chosen between 2 and 3, according to the abundance of introns in the respective species. Genes with homologous regions, but without the minimum number of windows with flanking introns, were classified as sequence shuffling (SS) genes.

**Figure 2:**
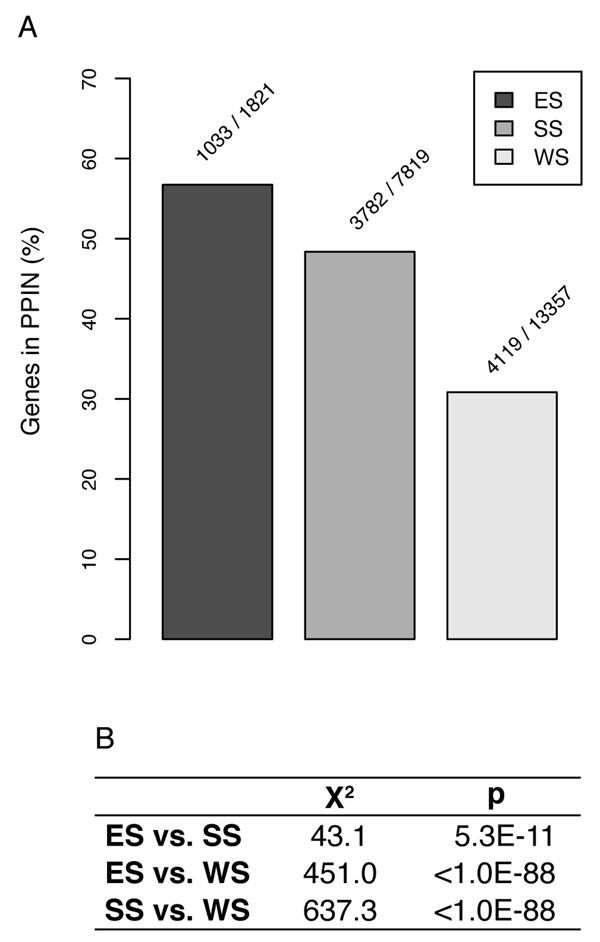
**Influence of shuffling upon presence in the human PPI network** (A) Percentage of protein-coding genes in the human PPI network according to shuffling profile category. Numbers above bars indicate the absolute number of genes in the network, and the total number of protein-coding genes of the species. (B) Chi-square values and p-values for comparisons among groups in Panel A concerning presence in PPI networks.

Based on the assumption that exon shuffling may be an important mechanism for the evolution of new protein-protein interactions, we first compared genes in the three categories according to their presence in PPI networks, as shown in Figure 2 (human) and Additional file 1 (remaining species). We observe that, in mammalian intron-rich species, there is a higher fraction of ES genes in PPI networks, compared to SS and WS genes. Interestingly, SS genes are also more frequently found in PPI networks than WS genes in all tested species, except worm.

**Figure 3:**
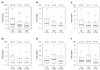
**Boxplot of vertex degree according to shuffling profile category for genes of several species** The analyzed species were human (A), mouse (B), *D. melanogaster* (C), *C. elegans* (D), *C. neoformans* (E), and *A. thaliana* (F). Only genes in the species PPI network are considered. For each group, protein length is reported as mean ± standard deviation. X and O indicate arithmetic and geometric mean, respectively. Whiskers mark percentiles 10 and 90.

We next performed pairwise comparisons of vertex degree among the three datasets. We opted for using multiple statistical measures, as each one could give us a particular appreciation of the differences present among the distributions of the several gene sets. Boxplots of the distributions including these measures are in Figure 3. We evaluated the statistical significance of the differences among groups by means of both U test, a standard non-parametric test for comparison of two samples, and re-sampling procedures for arithmetic and geometric means (in some cases, including a control for protein length, to rule out the possibility of degree differences to stem just from the known association between protein length and protein degree in PPI networks [17,29]). As seen in Table 1 (human) and in Additional file 2 (remaining species), the ES gene set has higher degree than the other two groups, according to all above-mentioned statistical tests, in all four analyzed metazoan species, but neither in fungus nor in plant. In addition, we observe that the SS set shows higher degree than the WS set in three cases: human, fungus and plant.

**Table 1:** Estimated p-values for comparisons of vertex degree among human gene groups shown in Figure 3A

RESAMPLING PROCEDURE									U TEST
**WITHOUT CONTROL FOR LENGTH**					**WITH CONTROL FOR LENGTH**				

	**Mean**		**Geom. Mean**		**Mean**		**Geom. Mean**		

	**p**	**Z**	**p**	**Z**	**p**	**Z**	**p**	**Z**	**p**

**ES vs. SS**	<3.0E-05	5.2	<3.0E-05	5.2	0.00051	3.9	<3.0E-05	4.5	3.6E-06
**ES vs. WS**	<3.0E-05	4.6	<3.0E-05	8.5	<3.0E-05	≥4.6	<3.0E-05	≥8.5	<6.6E-16
**SS vs. WS**	<3.0E-05	8.0	<3.0E-05	10.8	0.0012	3.5	<3.0E-05	8.2	

Z indicates difference between real data and the resampling mean given in standard deviations of the resampling mean.

We interpret all these results as follows: exon shuffling events are associated to the acquisition of new interactions by proteins, although its relative importance seems to be distinct among different phylogenetic groups. The resistance of this result to the use of both arithmetic and geometric mean in the re-sampling procedure, measures that give more or less weight to high-degree vertices, respectively, suggests additionally that this fact would apply to both low- and high-degree proteins.

SS genes probably represent a very heterogeneous set. They may correspond to homologous genes with sequence conservation limited to the currently identified homologous stretch or, given the widespread intron loss suffered along evolution by all analyzed species [27,30], to exon shuffling cases that lost too many introns and cannot be identified anymore. They may be examples of exon shuffling-generated genes in which sequence divergence in certain regions of the shuffled region prevented the expected correlation between introns and regions of sequence similarity to be found. Finally, they may correspond to genes resulting from all types of recombination events that are not mediated by introns.

Gene duplication is the other major evolutionary mechanism for the emergence of novelties, despite not being able to produce radically different genes in a short time span as exon shuffling can do. Is it known that eukaryotic genomes suffered both many single-gene and a few whole-genome duplications [31-35]. This has two consequences for our study. First, it is possible that a small number of extensively duplicated exon shuffling cases could explain all the degree differences observed among the shuffling profile categories. In order to rule this out, a paralog confluence procedure was performed in which sets of network vertices corresponding to paralogs were exchanged in the network by single vertices with degree equal to the average degree of the removed sets. After the confluence, in comparison to SS and WS sets, ES set continue to present higher degree, whichever the statistical test, with or without re-sampling control for length, in all four considered metazoan species, as shown in Additional file 2.

The second likely consequence of gene duplication for this study is the existence of a putative effect of gene duplications on PPI network vertex degree. To test that, the relationship between existence of paralogs of a gene and its corresponding vertex degree was analyzed. Given the strong effect we already know exon shuffling has upon degree for metazoan species, we excluded the exon shuffling genes before making the analysis for those species. As can be found in Additional file 3, we observed that genes with paralogs have a significantly higher degree in human, a significantly smaller degree in fly, and statistically non-significant differences in other organisms. It must be noticed that the quality of these data, contrary to the shuffling profile category ones, is not affected by intron loss, and probably must depend basically on the completeness of PPI network knowledge in the species. That knowledge is more extensive precisely in human and fly (see vertex degree average for each network in Methods), suggesting these opposing trends in the two species may be real. Most importantly, all degree differences between genes with and without paralogs are numerically smaller than the degree differences found between ES and non-ES gene sets, suggesting that gene duplication is not *per se* particularly important for the evolution of degree in PPI networks. It should be noted, however, that gene duplication may have an indirect importance as it is expected that exon shuffling would be less deleterious if involving duplicated copies of genes.

### Case analysis

A possible exon shuffling example promoting gain of a functionally relevant PPI may be furnished by amyloid precursor protein (APP). APP is present in vertebrates and has several isoforms resulting from alternative splicing, some of them (like APP751 and APP770) including an exon containing a Kunitz-type protease inhibitor (KPI) domain, which is absent from other isoforms (like APP695). APP usually suffers one out of two major types of processing by proteases: either it is cleaved by an alpha-secretase (usually the most active pathway), or is cleaved in succession by a beta- and a gamma-secretase [36,37]. Both pathways produce a soluble APP, although only the last one also leads to production of beta-amyloid peptide, which is an important component of amyloid plaques of Alzheimer disease and whose physiological function appears to be regulation of cellular cholesterol and sphingomyelin levels [38]. What determines the preferential cleavage pathway for APP is the presence or absence of the KPI domain, which inhibits alpha-secretase activity by binding to its trypsin domain. Besides that, soluble APP bearing the KPI domain also appears to favor beta amyloid accumulation in the CNS by other mechanisms [39,40]. The KPI domain of APP has long ago been proposed to have been acquired by exon shuffling [41]. Analyzing more recent sequence data, we found several facts to support this hypothesis: it is closely flanked by introns; some metazoan putative acceptor proteins, in organisms as diverse as protostomes, echinoderms and cephalochordates, display similarity to APP in regions both at 5' and 3' to the KPI domain, in a pattern that only requires a KPI domain insertion for production of a protein with domain structure very similar to that of APP (genes for these proteins never show sequences similar to KPI domain, making KPI domain loss or simple KPI domain alternative splicing less likely); conversely, some putative donor proteins, also widely distributed among metazoans, present the KPI domain flanked by introns, but not by any sequence presenting any similarity to other regions of APP.

### Exon shuffling and self-interactions

We next evaluated a special type of PPI, known as self-interaction, which refers to the capacity of a protein to interact with another copy of itself. The self-interacting proteins are noteworthy because they usually present higher degrees than non-self-interacting ones [42]. Indeed, for all species analyzed in this study, the set of self-interacting proteins have on average higher degree than the set of non-self-interacting proteins (data not shown). Besides that, self-interacting proteins are surprisingly abundant in the human and yeast PPI networks (24.6% and 25.0% of the proteins, respectively), and the duplication of self-interacting proteins probably has led to the evolution of many protein complexes [43,44].

Our three shuffling profile gene sets were compared in humans regarding their self-interaction properties. ES genes presented a higher frequency of self-interaction proteins than SS and WS genes (Table 2). This result resisted a statistical simulation with control for length as well as a paralog confluence procedure as described previously (Table 3, and Additional file 4), suggesting that exon shuffling events might promote the acquisition of self-interaction capacity in proteins.

**Table 2:** Percentage and explicit fraction of self-interacting human PPI network vertices according to shuffling profile category

	Self-interacting vertices
**ES**	31.8% (328/1033)
**SS**	24.5% (927/3782)
**WS**	22.8% (940/4119)

**Table 3:** Chi-square values and p-values regarding comparisons of self-interaction fractions among groups shown in Table 2

	CHI-SQUARE TEST		RESAMPLING PROCEDURE WITH CONTROL FOR LENGTH	
	**p**	**χ^2^**	**p**	**Z**

**ES vs. SS**	9.6E-06	21.7	<3.0E-05	5.1
**ES vs. WS**	1.0E-08	35.0	<3.0E-05	8.5
**SS vs. WS**	>0.05	3.0	**------**	**------**

Z indicates difference between real data and the resampling mean given in standard deviations of the resampling mean.

### Exon shuffling and interaction properties of domains

The observed relationship between exon shuffling and self-interactions led us to search for a mechanism by which exon shuffling might have promoted the emergence of self-interacting proteins. The gain of a domain capable of interacting with another copy of itself may be the simplest, most economical and most effective evolutionary solution to generate homodimeric proteins. We hypothesized that, if this mechanism had a significant participation in the evolution of the abundant self-interactions in the human PPI network, a stronger intron flanking signal (exon shuffling signal) would be expected to exist around self-interacting domains.

A number of studies have tried to disclosure what pairs of domains establish physical interactions, either based on structural data [45,46], or based on statistical methods applied to PPI networks enriched by domain data for each vertex [19,21,47,48,50]. Here, we simply analyzed the exon shuffling signal around domains that the literature usually classifies as self-interacting.

The relative frequency of intron flanking for self-interacting domains was compared to these relative frequencies for non-self-interacting domains that mediate PPIs and for domains that are not known to mediate PPIs. Again, in order to minimize the effect of gene duplication and concentrate on exon shuffling events, we used a paralog confluence procedure where the pondered contribution of each gene to the final average was given by the inverse of the number of domain-bearing paralogs of the gene. As seen in Tables 4 and 5, we observed a significant excess of introns flanking self-interacting domains in comparison to other types of domains classes in metazoan species as diverse as human and the sea anemone *Nematostella vectensis*. In fact, we found a similar pattern for all other analyzed metazoan species (data not shown). In contrast, for the choanoflagellate *Monosiga brevicollis* (phylogenetically very close to the metazoans [51]), as well as for more distant eukaryotic species such as *C. neoformans* and *A. thaliana* (Tables 4 and 5), this pattern was absent. These findings suggest that in the metazoan lineage (and probably since its emergence), self-interacting domains have suffered more exon shuffling when compared to non-self-interacting ones.

**Table 4:** Exon shuffling signal given by intron flanking for different classes of domain regarding self-interaction

Species	Group	Domain category	Flanked domains	Total domains	Flanking porcentage
***H. sapiens***	**I**	**Self-interacting**	3168.2	20357.3	15.6
	**II**	**Non-self-interacting DI**	180.5	1882.9	9.6
	**III**	**Non-DI**	548.8	4386.8	12.5

***N. vectensis***	**I**	**Self-interacting**	1078.2	10354.7	10.4
	**II**	**Non-self-interacting DI**	110.5	1591.4	6.9
	**III**	**Non-DI**	187.8	3532.0	5.3
					
***M. brevicollis***	**I**	**Self-interacting**	272.7	6798.0	4.0
	**II**	**Non-self-interacting DI**	29.8	951.8	3.1
	**III**	**Non-DI**	55.6	1946.4	2.9

***C. neoformans***	**I**	**Self-interacting**	98.5	3788.6	2.6
	**II**	**Non-self-interacting DI**	21.8	690.0	3.2
	**III**	**Non-DI**	34.8	1537.5	2.3

***A. thaliana***	**I**	**Self-interacting**	232.7	5575.0	4.2
	**II**	**Non-self-interacting DI**	29.9	813.5	3.7
	**III**	**Non-DI**	81.6	3760.5	2.2

DI = domain-interacting.

**Table 5:** Chi-square values and p-values for comparisons among groups shown in Table 4

Species	Group comparison	Chi-square	p
***H. sapiens***	**I vs. II**	47.6	1.5E-11
	**I vs. III**	26.1	9.6E-07
	**II vs. III**	10.7	3.3E-03

***N. vectensis***	**I vs. II**	18.1	6.3E-05
	**I vs. III**	81.9	4.2E-19
	**II vs. III**	5.0	>0.05

***M. brevicollis***	**I vs. II**	1.5	>0.05
	**I vs. III**	5.3	>0.05
	**II vs. III**	0.1	>0.05

***C. neoformans***	**I vs. II**	0.5	>0.05
	**I vs. III**	0.4	>0.05
	**II vs. III**	1.2	>0.05

***A. thaliana***	**I vs. II**	0.3	>0.05
	**I vs. III**	27.1	5.7E-07
	**II vs. III**	10.7	3.3E-03

Given the evidence that we found for the role of exon shuffling in the spread of self-interacting domains, we speculate about the evolutionary advantage that this type of exon shuffling might have. The gain by a protein of a domain that mediates PPIs might in principle make it possible for the acceptor protein to interact with proteins bearing the domains (and motifs) that interacted with the shuffled domain in the donor protein. We think that this kind of chemical affinity consequence is much more likely to have a functional consequence when the exon shuffling event involves self-interacting domains. Indeed, the abundance of self-interactions in humans and the widespread exon shuffling of self-interacting domains in metazoans may be molecular testimonies in favor of this hypothesis.

On the other hand, the same rationale would also predict that the gain of a domain by exon shuffling, whether self-interacting or not, would be more likely to have functional effects if the domain could interact with a higher number of other domains, leading us to expect a higher frequency of exon shuffling involving “promiscuous domain types”, that is, domain types that have the capacity to interact with many other domain types. We observed this to be true in all metazoan species that we analyzed, with data for some selected eukaryotic species seen in Tables 6 and 7. In contrast to the strong statistical significances seen for metazoan species (human and sea anemone), trends below the 0.05 significance level were also seen for choanoflagellate and plant, suggesting that similar phenomena might have taken place in non-metazoan eukaryotic species, although in much smaller scale, as attested by the rarity of domain intron flanking in those species.

**Figure 4:**
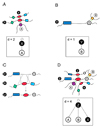
**An illustration of the effect upon the PPI network topology of an exon shuffling event(A) Protein X and its interaction partners.** The self-interacting red domain mediates two PPIs: a self-interaction of protein X and an interaction between proteins X and A. **(B) Protein Y and its interaction partners.** Protein Y has just one PPI, namely, with protein B. **(C) Exon shuffling event and evolution of a new protein.** Hypothetical exon shuffling event promotes the evolution of a new protein Z, merging together domains of proteins X and Y. **(D) Protein Z and its interaction partners.** The new protein Z may be able to interact with several proteins just by reuse of the interaction properties that its domains already possessed in proteins X and Y. In particular, protein Z has in this case become a self-interacting protein. "d" stands for vertex degree.

The observation that the exon shuffling gene set is enriched with promiscuous domains provides an explanation for the observed association between vertex degree and exon shuffling. An illustration of the effect upon the PPI network topology of an exon shuffling event leading to the gain of a domain with promiscuous property is seen in Figure 4. If a hypothetical exon shuffling event promotes the evolution of a new protein Z, merging together domains of proteins X and Y, this new protein Z may be able to interact with several proteins through the interaction properties already available in proteins X and Y. This suggests that exon shuffling events involving promiscuous domains could be responsible for the origin of hubs in PPI networks.

**Table 6:** Exon shuffling signal given by intron flanking for different classes of domain regarding promiscuity

Species	Group	Domain category	Flanked domains	Total domains	Flanking porcentage
***H. sapiens***	**I**	**High promiscuity DI**	2723.4	16080.7	16.9
	**II**	**Low promiscuity DI**	625.3	6159.4	10.2
	**III**	**Non-DI**	548.8	4386.8	12.5

***N. vectensis***	**I**	**High promiscuity DI**	904.5	6634.9	13.6
	**II**	**Low promiscuity DI**	284.2	5311.2	5.4
	**III**	**Non-DI**	187.8	3532.0	5.3

***M. brevicollis***	**I**	**High promiscuity DI**	197.2	4294.7	4.6
	**II**	**Low promiscuity DI**	105.2	3455.1	3.0
	**III**	**Non-DI**	55.6	1946.4	2.9

***C. neoformans***	**I**	**High promiscuity DI**	68.7	2264.9	3.0
	**II**	**Low promiscuity DI**	51.7	2213.7	2.3
	**III**	**Non-DI**	34.8	1537.5	2.3

***A. thaliana***	**I**	**High promiscuity DI**	152.3	3266.1	4.7
	**II**	**Low promiscuity DI**	110.4	3122.4	3.5
	**III**	**Non-DI**	81.6	3760.5	2.2

DI = domain-interacting.

**Table 7:** Chi-square values and p-values for comparisons among groups shown in Table 6

Species	Group comparison	Chi-square	p
***H. sapiens***	**I vs. II**	159.8	3.9E-36
	**I vs. III**	49.9	4.8E-12
	**II vs. III**	14.2	5.1E-04

***N. vectensis***	**I vs. II**	224.9	2.4E-50
	**I vs. III**	165.3	2.3E-37
	**II vs. III**	0.0	>0.05

***M. brevicollis***	**I vs. II**	11.8	1.8E-03
	**I vs. III**	9.9	4.8E-03
	**II vs. III**	0.1	>0.05

***C. neoformans***	**I vs. II**	1.8	>0.05
	**I vs. III**	1.8	>0.05
	**II vs. III**	0.0	>0.05

***A. thaliana***	**I vs. II**	4.9	>0.05
	**I vs. III**	33.0	2.8E-08
	**II vs. III**	11.2	2.4E-03

Another domain property that seems to be correlated to exon shuffling events is abundance (number of occurrences of a domain in a given proteome). It is already known that abundant domains have on average stronger intron flanking signal [10]. We observe this difference for metazoan species as diverse as sea anemone and human, but only marginally for choanoflagellate, and not at all for plant and fungus (Tables 8 and 9). Table 10 shows the number of human Pfam domain types present in categories for the following attributes: self-interaction and promiscuity; self-interaction and abundance; promiscuity and abundance. A significant association was found between all three attributes of domains, as can be seen in table 11. We initially observed that self-interacting domains are more promiscuous than expected by chance. Similarly, both self-interacting and promiscuous domains tend to be abundant domains. These associations were found to be strong for domains present in any analyzed eukaryotic species, despite the fact that the association of these characteristics to the intron flanking exon shuffling signal is strong only in metazoans. This could be due to the dynamics of intron evolution and shuffling phenomena in different species.

Cause and effect may be difficult to disentangle for the above-mentioned properties, and clearly more studies are necessary for this purpose. We speculate that the interaction properties of a domain are important in determining the probabilities for that domain to be spread by exon shuffling. In this sense, self-interaction and promiscuity would be properties to promote the abundance of domains along large evolutionary time frames.

**Table 8:** Exon shuffling signal given by intron flanking for different classes of domain regarding abundance

Species	Group	Domain category	Flanked domains	Total domains	Flanking porcentage
***H. sapiens***	**I**	**high abundance**	3729.5	22841.1	16.3
	**II**	**low abundance**	168.0	3785.9	4.4

***N. vectensis***	**I**	**high abundance**	1259.4	11899.5	10.6
	**II**	**low abundance**	117.0	3578.6	3.3

***M. brevicollis***	**I**	**high abundance**	278.4	7078.7	3.9
	**II**	**low abundance**	79.6	2617.5	3.0

***C. neoformans***	**I**	**high abundance**	98.6	3463.6	2.8
	**II**	**low abundance**	56.5	2552.5	2.2

***A. thaliana***	**I**	**high abundance**	243.6	7111.0	3.4
	**II**	**low abundance**	100.6	3038.0	3.3

**Table 9:** Chi-square values and p-values for comparisons between domain groups shown in Table 8

Species	Chi-square	p
***H. sapiens***	366.5	1.1E-81
***N. vectensis***	180.8	3.3E-41
***M. brevicollis***	4.0	4.5E-02
***C. neoformans***	2.1	>0.05
***A. thaliana***	0.1	>0.05

**Table 10:** Number of human Pfam domain types in categories for promiscuity, abundance and self-interactions

	**Promiscuous**	**Non-promiscuous**	**Abundant**	**Rare**

**Self-interacting**	303	936	385	854
**Non-self-interacting**	34	453	79	408

**Abundant**	160	287		
**Rare**	177	1102		

**Table 11:** Chi-square values and p-values for associations between domain properties seen in table 10

Association	Chi-square	p
**Self-interaction vs. Promiscuity**	66.8	3.0E-16
**Self-interaction vs. Abundance**	38.5	5.5E-10
**Promiscuity vs. Abundance**	83.9	5.1E-20

### Conclusions

In this work we have presented evidence for a relevant role of exon shuffling in the evolution of metazoan PPI networks. We also showed that some interaction properties of domains might make them more likely to take part into exon shuffling phenomena that promote the evolution of new genes bearing novel PPIs. This idea, added to the fact that metazoan exon shuffling genes specifically show higher degree, suggests that the role of exon shuffling in PPI networks is primarily to promote the creation of novel PPIs. Furthermore, the strong signal of exon shuffling in the set of promiscuous domains suggests a possible role of exon shuffling in the origin of hubs in PPI networks.

It is important to add a final remark concerning models normally used for PPI networks. Our findings suggest that an important evolutionary process contributing to the emergence of PPI networks of extant species has been overlooked in most graph growth models used for PPI network study in the literature. It has been repeatedly proposed that duplication models can explain the degree distribution of PPI networks [52-54], and even their subgraph distributions [55]. However, gene duplication does not usually lead to the evolution of neither longer proteins nor genes with an increased number of domains. This is in sharp contrast to exon shuffling, which has been shown to be commonly involved in the evolution of metazoan multidomain proteins [6]. Hence, any duplication model will not adequately predict the observed associations between degree and protein size, and between degree and number of domains [17,29].

Recently, a work has addressed the resilience of the powerlaw distribution produced by duplication models to shuffling phenomena interfering in the topology of the PPI network, and concluded that these phenomena did not significantly affect the powerlaw [54]. However, the shuffling model used in the mentioned work did not present any trend for increasing the degree of shuffled vertices, in disagreement with what we found evaluating real biological data in the present work. We think a natural next step in future investigations will be to define the relative contributions of duplication, exon shuffling and other shuffling events for the evolution of PPI networks in the several kingdoms of life, as well as to develop new graph growth models that better help us to understand the effects of these two evolutionary forces upon PPI network topology.

## Methods

### Sources of public data for protein-coding genes

We used information concerning aminoacid sequences and intron positions from Ensembl version 48, for *Homo sapiens*, *Mus musculus*, *Drosophila melanogaster*, and *Caenorhabditis elegans*, from JGI site, for *Nematostella vectensis* (filtered gene models; genome version 1.0) and *Monosiga brevicollis* (filtered gene models; genome version 1.0), and from NCBI site, for *Cryptococcus neoformans* (*var. neoformans JEC21*; August 2008 version) and for *Arabidopsis thaliana* (December 2007 version). Protein domain occurrences were identified using Pfam A (release 22), and an e-value smaller than 1e-2 was required for an occurrence to be accepted.

### PPI networks

Given the existence of several incomplete public primary PPI databases with different standards, we adopted the common practice of merging their data [22,56-58]. The following databases were used: MINT (December 2007 version), BIOGRID (2.0.37), INTACT (January 2008 version), HPRD (September 2007 version), BIND (May 2006 version), DIP (January 2008 version). However, in PPI networks for *S. cerevisiae* (and the resulting projected PPI network of *C. neoformans*; see below) and *A. thaliana*, more recent versions of some databases were used (MINT version of October 2008, BIOGRID version 2.0.46, INTACT version of November 2008, DIP version of October 2008), because we verified that updates in these versions were particularly relevant for these species, but not for the remaining ones. For all species, these databases include both high-throughput experiments and low-throughput ones curated from the literature. Several filters were adopted to reduce possible noise sources. When a description of the technique used to discover the interaction was available, those registers with an entry that referred to one of the several mass spectrometry-based methods were excluded, in order to avoid including indirect interactions. Exclusively functional interactions, for example numerous exclusively genetic interactions present in BIOGRID, were also excluded. Conversion from Refseq and Uniprot protein identifiers and Ensembl and species-specific gene identifiers to Entrez gene identifiers were made using conversion tables available in the Gene section of NCBI site (tables gene2accession, gene_info), with some conversions also using tables uniprot_trembl.dat and uniprot_sprot.dat available at the Uniprot site. The used versions for these four conversion tables were of November 2008 (for *S. cerevisiae* and *A. thaliana*) or February 2008 versions (for the remaining analyzed species). For the metazoan networks, vertex degree was attributed based on a network were each vertex corresponded to a unique Entrez Gene ID; also, vertices with degree equal to zero were discarded. Given the comprehensive conversion tables for protein identifiers made available by NCBI (tables gene2accession, gene_info), we considered that this would be a good strategy for maximizing the use of information present in the six raw networks for the process of degree estimation. By means of this pipeline, we obtained networks for *H. sapiens* (9897 vertices e 41981 edges), *D. melanogaster* (7647 vertices e 25988 edges), *C. elegans* (3191 vertices e 5295 edges), *M. musculus* (2565 vertices e 3302 edges), and *A. thaliana* (1283 vertices and 2381 edges). However, for the purposes of analyzing shuffling profile categories in metazoan species, we considered only those vertices with a corresponding Ensembl Gene ID in the gene_info NCBI table, after what remained: 8934 vertices (degree average 8.3) for *H. sapiens*; 2444 vertices (degree average 2.5) for *M. musculus*; 7576 vertices (average degree 6.8) for *D. melanogaster*; 3186 vertices (average degree 3.3) for *C. elegans*. For *Cryptococcus neoformans*, a PPI network based on Entrez Gene ID was obtained as described above for *Saccharomyces cerevisiae*, and the Inparanoid algorithm [59] was used to generate a network where each pair of *C. neoformans* genes will interact if and only if their corresponding *S. cerevisiae* orthologs also do. From this procedure resulted a network containing 2395 vertices, with degree average equal to 16.0. For both *C. neoformans* and *A. thaliana*, shuffling gene categories analysis was made using RefSeq identifiers. All network genes had a corresponding maximal length Ensembl or RefSeq protein, and the length of this protein was also considered to be the protein length of the network vertex.

### Gene sets enriched in exon and sequence shuffling events

At first, we considered the set of all protein-coding genes for the species under analysis. For each gene, we just considered one protein of maximal length. Using Blastp 2.2.17, local alignments were performed between any two proteins, and putatively homologous regions in non-homologous genes were identified by the following criteria: alignments that presented e-value less than 1e-3, and covered less than 50% of the shorter protein. We sought for the presence of introns in the four windows flanking the aligned coding sequence. Counting in relation to the extremities of the aligned region, each window extended from the third nucleotide toward the region interior to the twentieth nucleotide toward the region exterior. We required that at least three of these windows to contain introns for *H. sapiens* and *M. musculus*. For *D. melanogaster*, *C. elegans*, *A. thaliana* and *C. neoformans*, species with more extensive intron loss, just two windows with introns were required. Regardless of which genes presented the flanking introns, any two genes whose corresponding aligned proteins met the above requirement were considered to belong to the exon shuffling (ES) gene set. Genes with aligned regions that met the homology criteria but such that none of these regions had the adequate intron flanking were classified as sequence (SS) genes, if they had not been included in the ES set the previous step. The remaining genes, without any homologous region to a non-homologous gene, were classified as part of the without-shuffling (WS) gene set.

### Paralogy assessment

When possible, paralogy was defined through lists made publicly available in Ensembl site. For the species for which this was not possible, we made all-against-all Blastp 2.2.17 alignments of proteins of maximal length from each protein-coding gene and defined as paralogous any two genes that had an alignment with added non-superimposable high-scoring sequence pairs comprising at least 70% of the length of the longest of the two proteins. We also considered paralogy to be a transitive property: if gene A is paralogous to gene B, and gene B is paralogous to gene C, then gene A is also paralogous to gene C.

### Paralogous gene confluence procedure for PPI networks

Comparisons that evidenced statistically significant differences among ES, SS and WS gene sets were subjected to a confirmatory procedure to rule out the possibility of the result to be due only to an unanticipated expansion of some gene families through gene duplication. The procedure consisted in making a confluence of all sets of genes that were paralogous among themselves and that belonged to the same of the three shuffling sets (genes in distinct sets were not merged). Each subset of vertices corresponding to these paralogous genes was replaced by a single new vertex with degree and protein length equal to the average degree and average protein length of the subset, respectively.

### Statistical analyses for degree in PPI networks in selected gene sets

For analyzing association between gene presence in PPI networks and shuffling profile categories, we used chi-square test with correction for continuity. For evaluating degree distribution among the ES, SS, and WS sets, because of the strongly asymmetric and non-gaussian nature the data, we made use of the following comparison techniques: resampling procedure for estimating p-value for the arithmetic and geometric degree mean differences, with or without a control for length; U test. Given the fact that we made three tests for each species (ES vs. SS, ES vs. WS, SS vs. WS), in all above-mentioned cases we conservatively used a Bonferroni correction. The resampling procedure without control for length consisted of just randomly selecting samples with the size of the smaller of two sets under comparison from the union of these sets, and counting the fraction of samples that had more extreme degree arithmetic/geometric means than the ones present in the real data. The alternative hypothesis assumed that these means were different, so this fraction was multiplied by 2. The control for length in the resampling procedure involved making a one-to-one correspondence between vertices of the real sets and vertices of randomly selected sets, restricting the random selection of each vertex to a set of vertices with protein length average equal to the protein length of a corresponding vertex in the real set. This assured that the randomly drawn sets had protein length averages approximately equal to the real set under analysis. Similar resamplig procedures were used for investigating the association between shuffling profile categories and protein self-interactions, and the association between vertex degree in PPI networks and gene duplication.

### Intron flanking of domains

We sought for the presence of introns in the two windows flanking the domains.Counting from the domain extremities, each window extended from the third nucleotide toward the domain interior to the thirty-second nucleotide toward its exterior. We required both windows to contain at least one intron each. A paralog confluence procedure was also used when counting the number of both total and flanked domains: a domain in a gene containing k paralogs in the genome was counted as if it was only 1/k of a domain.

### Interaction properties of domains

Possible domain-domain interactions were obtained from DOMINE (version 1.1 of February 2008). Only structural and high confidence predicted interactions were considered. Given this selected set of domain-domain interactions, Pfam domain types were arbitrarily classified in three categories: promiscuous domain-interacting domains, if they had six or more domain-domain interactions, non-promiscuous domain-interacting domains, if they had between one and five domain-domain interactions, and non-domain-interacting domains, if they had no domain-domain interaction. This same selected set was used to define if the domain-interacting domains (those with at least one domain-domain interaction) were self-interacting or not.

### Domain abundance

Domains types were arbitrarily classified as having “low abundance” or “high abundance” if their total number of occurrences in the genome was up to three, or four or more, respectively.

### Association between domain properties

The association between domain properties (intron flanking, promiscuity, abundance and self-interaction) was statistically evaluated by chi-square test with correction for continuity. When multiple tests were performed (tables 5 and 7), p-values were multiplied by the number of tests, which is equivalent to applying the Bonferroni correction.

## Competing interests

The authors declare that they have no competing interests.

## Authors’ contributions

DVC wrote most of the code and drafted the manuscript. GSF wrote the code for identifying exon shuffling cases by means of sequence alignment and created the illustrations. SJS conceived the investigation, revised the manuscript and supervised the work. All authors read and approved the final manuscript.

## Supplementary Material

Additional file 1 **Influence of shuffling upon presence in PPI networks in selected species**. Percentage of protein-coding genes in PPI networks according to shuffling profile category in mouse, worm, fly, fungus, and plant species (bar charts), and chi-square values and p-values for comparisons among shuffling profile groups concerning presence in PPI networks (tables). Numbers above bars indicate the absolute number of genes in the corresponding PPI network, and the total number of protein-coding genes of the species. Click here for file

Additional file 2 **Vertex degree in PPI networks according to shuffling profile category for selected species**. Data come from the following species: human (A/E), mouse (F and B/G), *D. melanogaster* (H and C/I), *C. elegans* (J and D/K), *C. neoformans* (L), and *A. thaliana* (M). Boxplots and tables display, respectively, vertex degree distributions and p-values for group comparisons. Tables F, H, J, L, and M make use of all genes in PPI network, and present the results of statistical analyses concerning main text figures 3B, 3C, 3D, 3E and 3F, respectively. In contrast, boxplot/table pairs A/E, B/G, C/I, and D/K consider genes in PPI network after a paralog confluence procedure for vertices in order to control for the effect of gene duplications. In boxplots, X and O indicate arithmetic and geometric mean, respectively, whiskers mark percentiles 10 and 90, and protein length for each group is reported as mean ± standard deviation. Click here for file

Additional file 3 **Vertex degree in PPI networks for genes with and without paralogs**. In boxplots of vertex degree, analyzed species were human (A), mouse (B), *D. melanogaster* (C), *C. elegans* (D), *C. neoformans* (E), and *A. thaliana* (F). In metazoan species (A to D), genes of the ES subsets were previously excluded. Estimated p-values for group comparisons are found in tables G (human and mouse), H (fly and worm) and I (fungus and plant). In boxplots, X and O indicate arithmetic and geometric mean, respectively, whiskers mark percentiles 10 and 90, and protein length for each group is reported as mean ± standard deviation. Click here for file

Additional file 4 **Self-interactions according to shuffling profile category in human**. Percentages and explicit fractions of self-interacting vertices, for the human PPI network subjected to a paralog confluence procedure, are seen in part 4A, whereas chi-square values and p-values regarding comparisons among groups are shown in part 4B. Click here for file
