# Supplementary material for: The role of exon shuffling in shaping protein-protein interaction networks
Source: BMC Genomics. 2010 Dec 22;11(Suppl 5):S11. doi: 10.1186/1471-2164-11-S5-S11 (PMC3045794; doi:10.1186/1471-2164-11-S5-S11)
Supplement: Additional file 1 — Influence of shuffling upon presence in PPI networks in selected species. Percentage of protein-coding genes in PPI networks according to shuffling profile category in mouse, worm, fly, fungus, and plant species (bar charts), and chi-square values and p-values for comparisons among shuffling profile groups concerning presence in PPI networks (tables). Numbers above bars indicate the absolute number of genes in the corresponding PPI network, and the total number of protein-coding genes of the species. [file 1471-2164-11-S5-S11-S1.pdf]

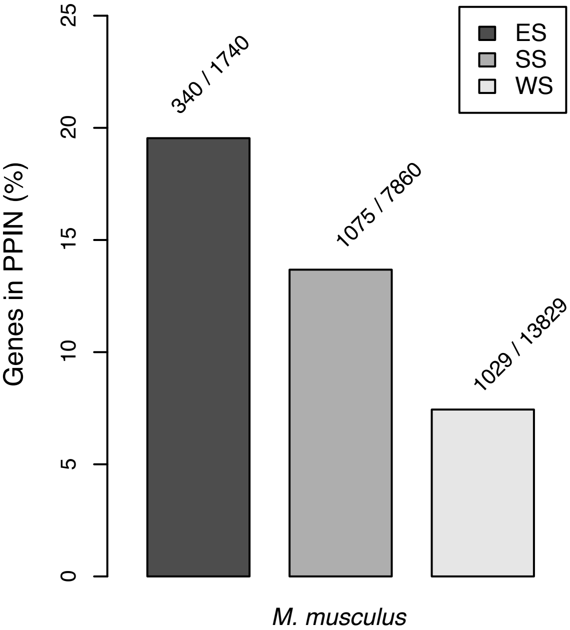

Genes in PPIN (%)
0
5
10
15
20
25
340 / 1740
1075 / 7860
1029 / 13829
ES
SS
WS
M. musculus

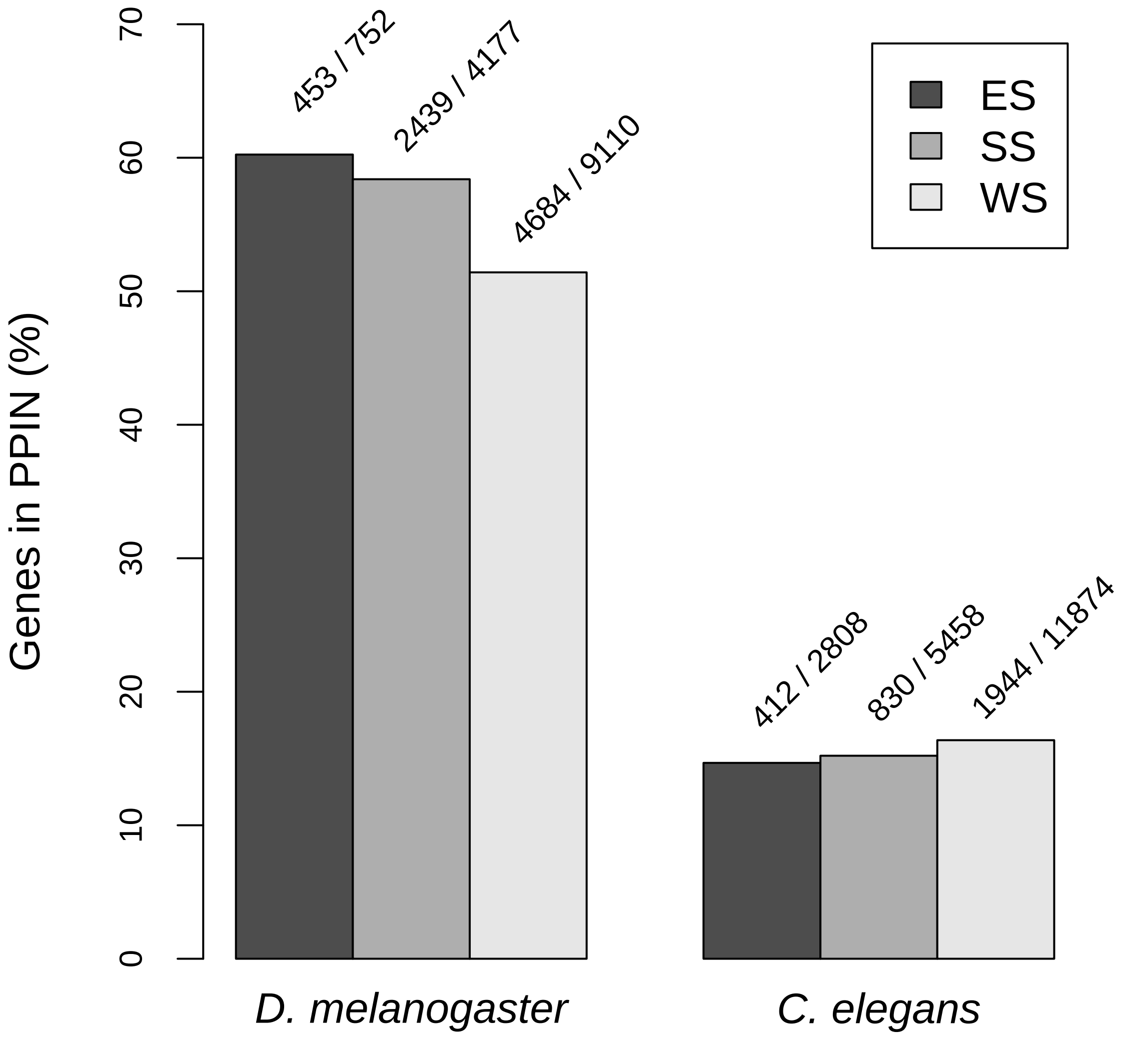

Genes in PPIN (%)
0
10
20
30
40
50
60
70
453 / 752
2439 / 4177
4684 / 9110
412 / 2808
830 / 5458
1944 / 11874
ES
SS
WS
D. melanogaster
C. elegans

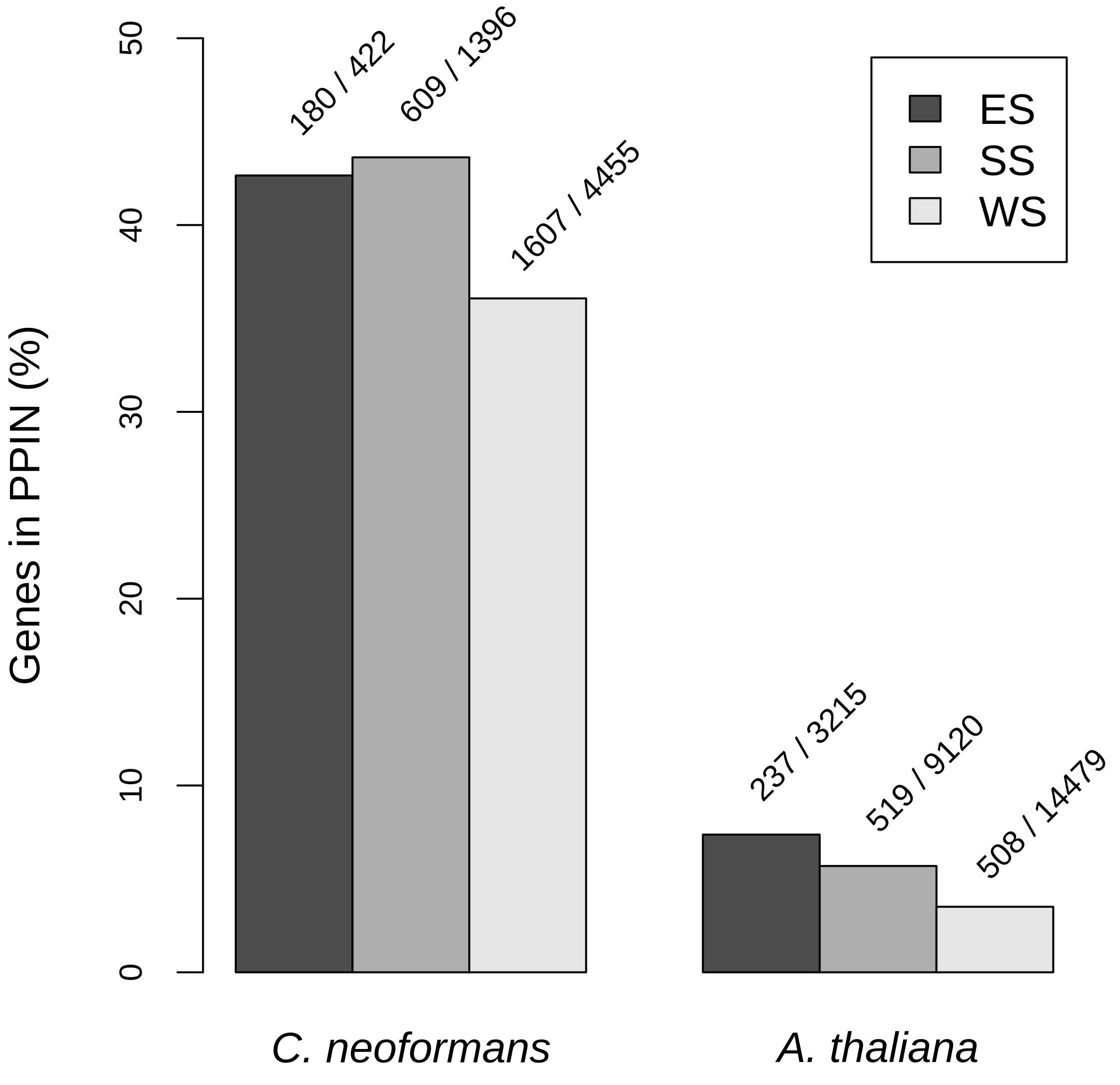

Genes in PPIN (%)
0
10
20
30
40
50
180 / 422
609 / 1396
1607 / 4455
237 / 3215
519 / 9120
508 / 14479
ES
SS
WS
C. neoformans
A. thaliana

	M .musculus		*D. melanogaster*		*C. elegans*	
	**χ2**	**p**	**χ2**	**p**	**χ2**	**p**
**ES vs. SS**	51.8	1.9E-12	0.8	>0.05	0.0	>0.05
**ES vs. WS**	240.9	7.5E-54	21.4	1.1E-05	3.1	>0.05
**SS vs. WS**	207.9	1.2E-46	55.8	2.4E-13	3.7	>0.05

	C. neoformans		*A. thaliana*	
	**χ2**	**p**	**χ2**	**p**
**ES vs. SS**	0.1	>0.05	11.4	2.2E-03
**ES vs. WS**	6.9	0.026	96.4	2.7E-22
**SS vs. WS**	25.4	1.4E-06	63.5	4.8E-15
