# Supplementary material for: The role of exon shuffling in shaping protein-protein interaction networks
Source: BMC Genomics. 2010 Dec 22;11(Suppl 5):S11. doi: 10.1186/1471-2164-11-S5-S11 (PMC3045794; doi:10.1186/1471-2164-11-S5-S11)
Supplement: Additional file 2 — Vertex degree in PPI networks according to shuffling profile category for selected species. Data come from the following species: human (A/E), mouse (F and B/G), D. melanogaster (H and C/I), C. elegans (J and D/K), C. neoformans (L), and A. thaliana (M). Boxplots and tables display, respectively, vertex degree distributions and p-values for group comparisons. Tables F, H, J, L, and M make use of all genes in PPI network, and present the results of statistical analyses concerning main text figures 3B, 3C, 3D, 3E and 3F, respectively. In contrast, boxplot/table pairs A/E, B/G, C/I, and D/K consider genes in PPI network after a paralog confluence procedure for vertices in order to control for the effect of gene duplications. In boxplots, X and O indicate arithmetic and geometric mean, respectively, whiskers mark percentiles 10 and 90, and protein length for each group is reported as mean ± standard deviation. [file 1471-2164-11-S5-S11-S2.pdf]

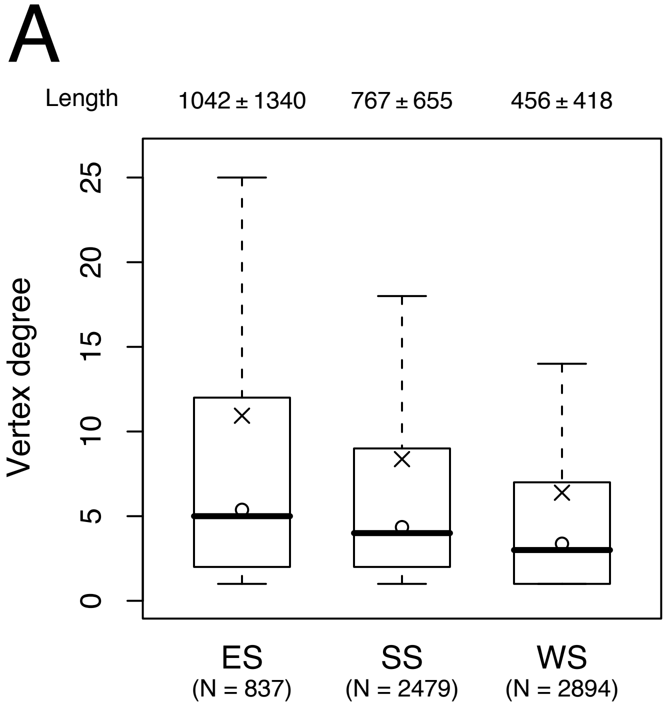
A
Length
1042 ± 1340
767 ± 655
456 ± 418
Vertex degree
0
5
10
15
20
25
ES
(N = 837)
SS
(N = 2479)
WS
(N = 2894)

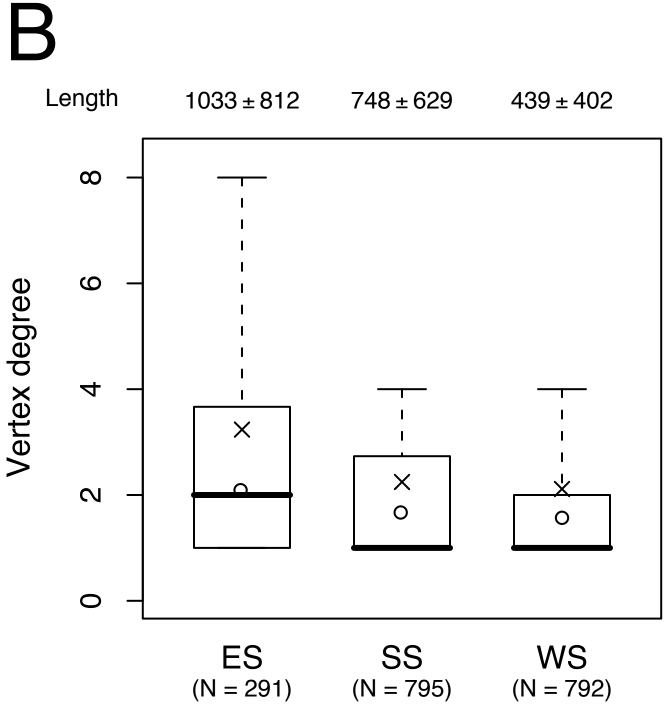
B
Length
1033 ± 812
748 ± 629
439 ± 402
Vertex degree
0
2
4
6
8
ES
(N = 291)
SS
(N = 795)
WS
(N = 792)

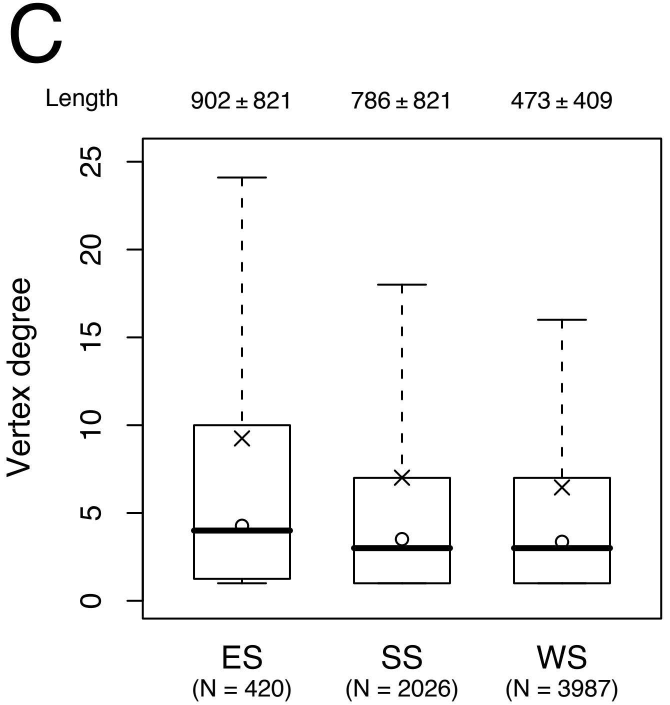
C
Length
902 ± 821
786 ± 821
473 ± 409
Vertex degree
0
5
10
15
20
25
ES
(N = 420)
SS
(N = 2026)
WS
(N = 3987)

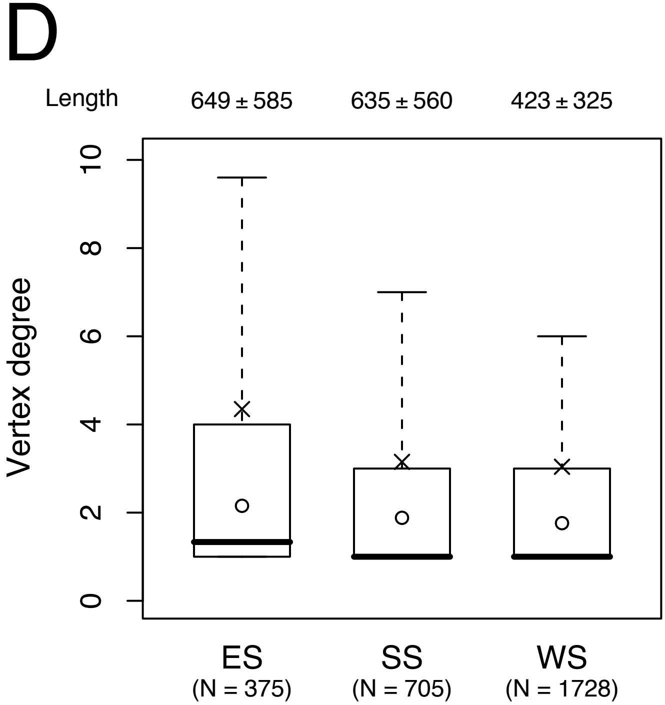

D
Length
649 ± 585
635 ± 560
423 ± 325
Vertex degree
0
2
4
6
8
10
ES
(N = 375)
SS
(N = 705)
WS
(N = 1728)

E

	PARALOG CONFLUENCE								
	RESAMPLING PROCEDURE								U TEST
	WITHOUT CONTROL FOR LENGTH				WITH CONTROL FOR LENGTH				
	Mean		Geom. Mean		Mean		Geom. Mean		
	p	Z	p	Z	p	Z	p	Z	p
**ES vs. SS**	<6.0E-06	4.3	3.0E-05	4.7	0.0012	3.5	0.00042	3.9	0.0018
**ES vs. WS**	<6.0E-06	9.0	<6.0E-06	10.5	<6.0E-05	6.1	<6.0E-05	8.5	1.1E-08
**SS vs. WS**	<3.0E-05	5.9	<3.0E-05	8.2	<1.2E-04	5.6	<1.2E-04	≥8.2	0.0014

Z indicates difference between real data and the resampling mean given in standard deviations of the resampling mean.

F

ALL GENES IN THE NETWORK									
	RESAMPLING PROCEDURE								U TEST
	WITHOUT CONTROL FOR LENGTH				WITH CONTROL FOR LENGTH				
	Mean		Geom. Mean		Mean		Geom. Mean		
	p	Z	p	Z	p	Z	p	Z	p
**ES vs. SS**	<3.0E-05	5.3	<3.0E-05	4.6	<3.0E-05	≥5.3	<3.0E-05	≥4.6	0.0017
**ES vs. WS**	<3.0E-05	6.0	<3.0E-05	5.9	<3.0E-05	≥6.0	<3.0E-05	≥5.9	1.1E-06
**SS vs. WS**	>0.05	1.2	>0.05	2.2	-------	-------	-------	-------	0.026

Z indicates difference between real data and the resampling mean given in standard deviations of the resampling mean.

G

	PARALOG CONFLUENCE								
	RESAMPLING PROCEDURE								U TEST
	WITHOUT CONTROL FOR LENGTH				WITH CONTROL FOR LENGTH				
	Mean		Geom. Mean		Mean		Geom. Mean		
	p	Z	p	Z	p	Z	p	Z	p
**ES vs. SS**	<3.0E-05	5.2	0.00033	4.0	<3.0E-05	≥5.2	≤0.00033	≥4.0	0.021
**ES vs. WS**	<3.0E-05	5.7	<3.0E-05	5.5	<3.0E-05	≥5.7	<3.0E-05	≥5.5	1.9E-05
**SS vs. WS**	>0.05	1.1	>0.05	2.3	-------	-------	-------	-------	0.012

Z indicates difference between real data and the resampling mean given in standard deviations of the resampling mean.

H

ALL GENES IN THE NETWORK									
	RESAMPLING PROCEDURE								U TEST
	WITHOUT CONTROL FOR LENGTH				WITH CONTROL FOR LENGTH				
	Mean		Geom. Mean		Mean		Geom. Mean		
	p	Z	p	Z	p	Z	p	Z	p
**ES vs. SS**	2.7E-03	3.5	3.9E-03	3.3	3.9E-03	3.4	4.2E-03	3.2	0.030
**ES vs. WS**	1.8E-04	4.8	6.0E-05	4.3	≤1.8E-04	4.8	6.0E-05	4.3	9.3E-04
**SS vs. WS**	>0.05	1.7	>0.05	1.6	-------	-------	-------	-------	>0.05

Z indicates difference between real data and the resampling mean given in standard deviations of the resampling mean.

I

PARALOG CONFLUENCE									
	RESAMPLING PROCEDURE								U TEST
	WITHOUT CONTROL FOR LENGTH				WITH CONTROL FOR LENGTH				
	Mean		Geom. Mean		Mean		Geom. Mean		
	p	Z	p	Z	p	Z	p	Z	p
**ES vs. SS**	2.7E-03	3.6	>0.05	3.0	≤0.0027	≥3.6	-------	-------	9.3E-03
**ES vs. WS**	1.2E-05	5.1	5.4E-05	4.3	≤1.2E-05	≥5.1	≤5.4E-05	≥4.3	4.8E-04
**SS vs. WS**	>0.05	2.0	>0.05	2.0	-------	-------	-------	-------	>0.05

Z indicates difference between real data and the resampling mean given in standard deviations of the resampling mean.

J

ALL GENES IN THE NETWORK									
	RESAMPLING PROCEDURE								U TEST
	WITHOUT CONTROL FOR LENGTH				WITH CONTROL FOR LENGTH				
	Mean		Geom. Mean		Mean		Geom. Mean		
	p	Z	p	Z	p	Z	p	Z	p
**ES vs. SS**	0.024	2.8	>0.05	2.2	>0.05	2.0	-------	-------	>0.05
**ES vs. WS**	0.040	2.9	2.4E-03	3.5	≤0.040	≥2.9	>0.05	2.9	0.026
**SS vs. WS**	>0.05	0.0	>0.05	1.3	-------	-------	-------	-------	>0.05
**ES vs. SS + WS**	0.022	3.2	4.4E-03	3.3	≤0.022	≥3.2	0.044	2.6	0.056

Z indicates difference between real data and the resampling mean given in standard deviations of the resampling mean.

K

PARALOG CONFLUENCE									
	RESAMPLING PROCEDURE								U TEST
	WITHOUT CONTROL FOR LENGTH				WITH CONTROL FOR LENGTH				
	Mean		Geom. Mean		Mean		Geom. Mean		
	p	Z	p	Z	p	Z	p	Z	p
**ES vs. SS**	0.032	2.7	>0.05	2.4	>0.05	1.7	-------	-------	>0.05
**ES vs. WS**	0.040	2.9	2.8E-04	4.3	≤0.040	≥2.9	3.2E-04	4.1	3.2E-03
**SS vs. WS**	>0.05	0.3	>0.05	1.9	-------	-------	-------	-------	>0.05
**ES vs. SS + WS**	0.028	3.1	4.4E-04	3.9	≤0.028	≥3.1	9.8E-03	3.1	0.010

Z indicates difference between real data and the resampling mean given in standard deviations of the resampling mean.

L

ALL GENES IN THE NETWORK									
	RESAMPLING PROCEDURE								U TEST
	WITHOUT CONTROL FOR LENGTH				WITH CONTROL FOR LENGTH				
	Mean		Geom. Mean		Mean		Geom. Mean		
	p	Z	p	Z	p	Z	p	Z	p
**ES vs. SS**	>0.05	-2.1	>0.05	-1.6	-------	-------	-------	-------	>0.05
**ES vs. WS**	>0.05	-0.6	>0.05	0.7	-------	-------	-------	-------	>0.05
**SS vs. WS**	0.0057	3.2	1.4E-04	4.1	≤0.0057	≥3.2	3.6E-04	3.6	1.6E-04

Z indicates difference between real data and the resampling mean given in standard deviations of the resampling mean.

M

ALL GENES IN THE NETWORK									
	RESAMPLING PROCEDURE								U TEST
	WITHOUT CONTROL FOR LENGTH				WITH CONTROL FOR LENGTH				
	Mean		Geom. Mean		Mean		Geom. Mean		
	p	Z	p	Z	p	Z	p	Z	p
**ES vs. SS**	>0.05	-1.9	>0.05	-1.9	-------	-------	-------	-------	>0.05
**ES vs. WS**	>0.05	1.5	3.9E-04	3.9	-------	-------	≤3.9E-04	≥3.9	6.9E-05
**SS vs. WS**	4.8E-05	4.1	<1.2E-03	6.9	≤4.8E-05	≥4.1	≤1.2E-03	≥6.9	2.4E-12

Z indicates difference between real data and the resampling mean given in standard deviations of the resampling mean.
