# Supplementary material for: The role of exon shuffling in shaping protein-protein interaction networks
Source: BMC Genomics. 2010 Dec 22;11(Suppl 5):S11. doi: 10.1186/1471-2164-11-S5-S11 (PMC3045794; doi:10.1186/1471-2164-11-S5-S11)
Supplement: Additional file 3 — Vertex degree in PPI networks for genes with and without paralogs. In boxplots of vertex degree, analyzed species were human (A), mouse (B), D. melanogaster (C), C. elegans (D), C. neoformans (E), and A. thaliana (F). In metazoan species (A to D), genes of the ES subsets were previously excluded. Estimated p-values for group comparisons are found in tables G (human and mouse), H (fly and worm) and I (fungus and plant). In boxplots, X and O indicate arithmetic and geometric mean, respectively, whiskers mark percentiles 10 and 90, and protein length for each group is reported as mean ± standard deviation. [file 1471-2164-11-S5-S11-S3.pdf]

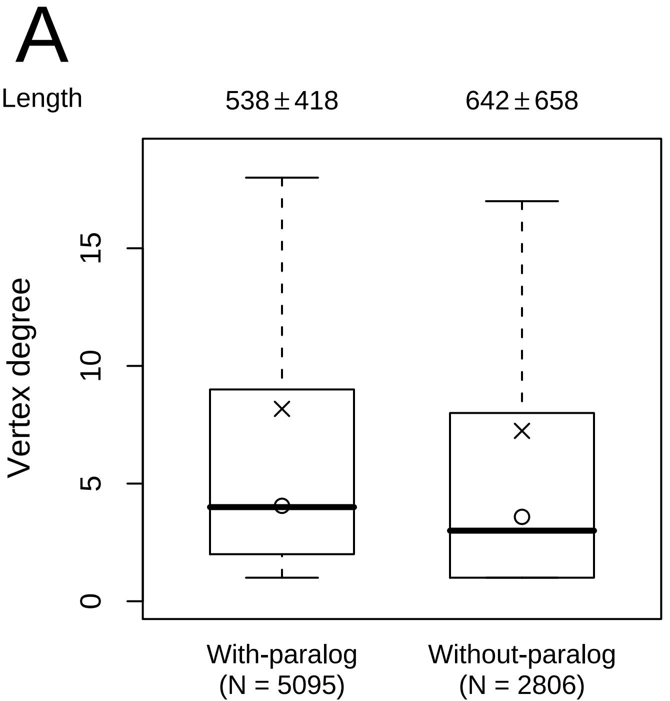
A
Length
538 ± 418
642 ± 658
Vertex degree
0
5
10
15
With-paralog
(N = 5095)
Without-paralog
(N = 2806)

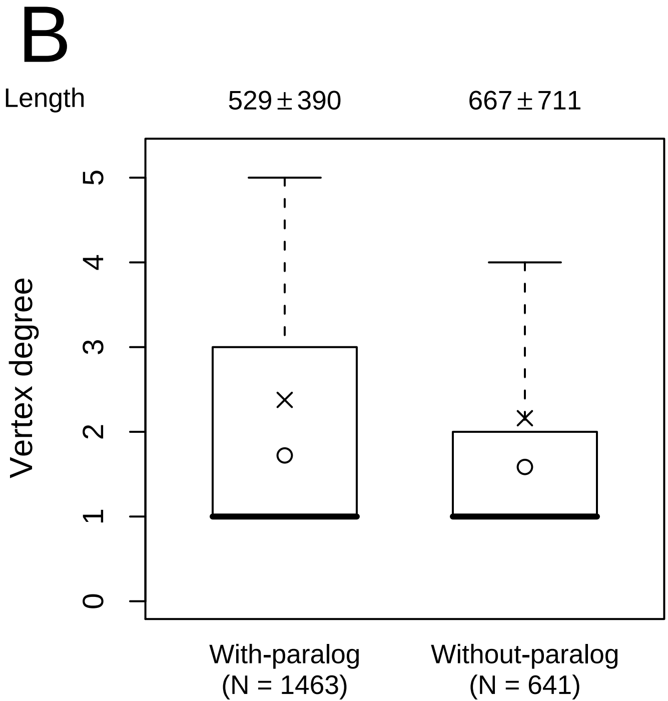
B
Length
529 ± 390
667 ± 711
Vertex degree
0
1
2
3
4
5
With-paralog
(N = 1463)
Without-paralog
(N = 641)

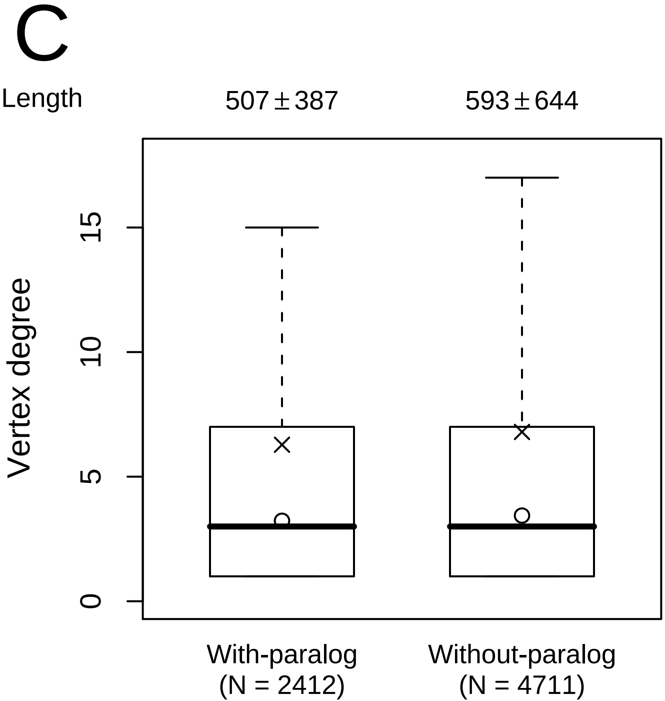

C
Length
507 ± 387
593 ± 644
Vertex degree
0
5
10
15
With-paralog
(N = 2412)
Without-paralog
(N = 4711)

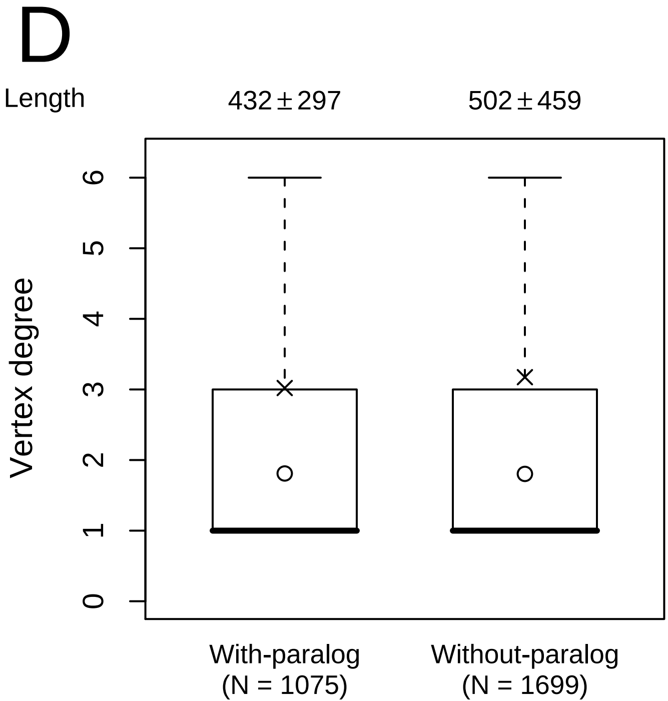
D
Length
432±297
502±459
Vertex degree
0
1
2
3
4
5
6
With-paralog
(N = 1075)
Without-paralog
(N = 1699)

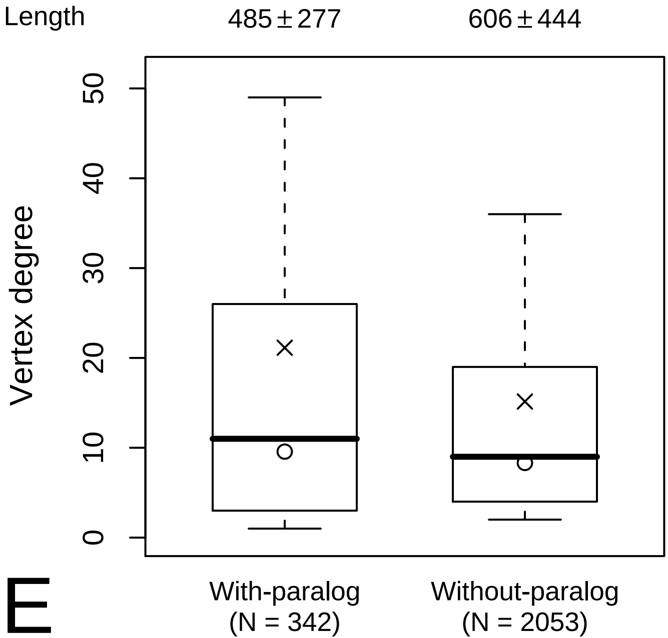
Length
485 ± 277
606 ± 444
Vertex degree
0
10
20
30
40
50
With-paralog
(N = 342)
Without-paralog
(N = 2053)
E

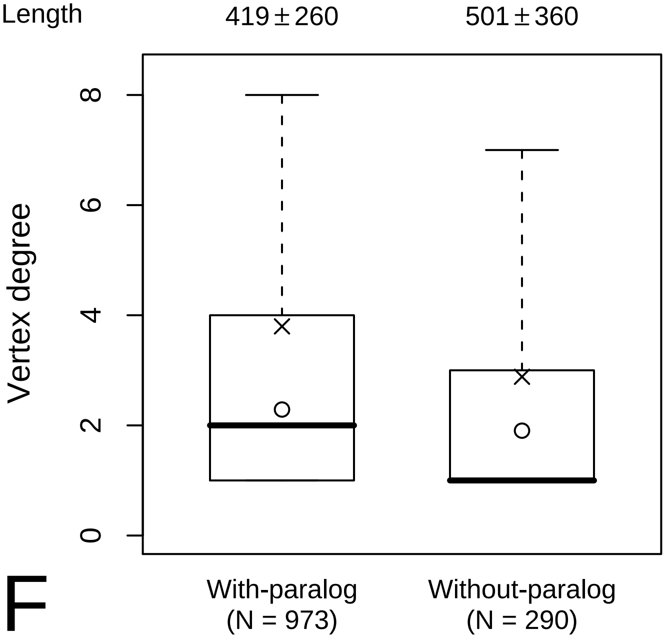
Length
419 ± 260
501 ± 360
Vertex degree
0
2
4
6
8
With-paralog
(N = 973)
Without-paralog
(N = 290)
F

G

| | RESAMPLING PROCEDURE | | | | U TEST |
|---|---|---|---|---|---|
| | Mean | | Geom. Mean | | |
| | p | Z | p | Z | |
| ***H. sapiens*** | 0.0043 | 2.8 | <2.0E-06 | 4.7 | 1.5E-06 |
| ***M. musculus*** | >0.05 | 1.7 | 0.011 | 2.5 | 0.004 |

Z indicates difference between real data and the resampling mean given in standard deviations of the resampling mean.

H

| | RESAMPLING PROCEDURE | | | | U TEST |
|---|---|---|---|---|---|
| | **Mean** | | **Geom. Mean** | | |
| | **p** | **Z** | **p** | **Z** | **p** |
| ***D. melanogaster*** | 0.044 | -2.0 | 0.019 | -2.3 | 0.025 |
| ***C. elegans*** | >0.05 | -0.6 | >0.05 | 0.1 | >0.05 |

Z indicates difference between real data and the resampling mean given in standard deviations of the resampling mean.

I

| | RESAMPLING PROCEDURE | | | | U TEST |
|---|---|---|---|---|---|
| | Mean | | Geom. Mean | | |
| | p | Z | p | Z | p |
| ***C. neoformans*** | 2.2E-05 | 4.7 | 0.022 | 2.3 | 0.022 |
| ***A. thaliana*** | 3.7E-04 | 3.1 | 1.9E-04 | 3.6 | 3.2E-04 |

Z indicates difference between real data and the resampling mean given in standard deviations of the resampling mean.
