# Supplementary material for: The role of exon shuffling in shaping protein-protein interaction networks
Source: BMC Genomics. 2010 Dec 22;11(Suppl 5):S11. doi: 10.1186/1471-2164-11-S5-S11 (PMC3045794; doi:10.1186/1471-2164-11-S5-S11)
Supplement: Additional file 4 — Self-interactions according to shuffling profile category in human. Percentages and explicit fractions of self-interacting vertices, for the human PPI network subjected to a paralog confluence procedure, are seen in part 4A, whereas chi-square values and p-values regarding comparisons among groups are shown in part 4B. [file 1471-2164-11-S5-S11-S4.pdf]

4A

	Self-interacting vertices
**ES**	34.2% (286/837)
**SS**	29.8% (738/2479)
**WS**	25.4% (736/2894)

4B

PARALOG CONFLUENCE				
	CHI-SQUARE TEST		RESAMPLING PROCEDURE WITH CONTROL FOR LENGTH	
	**p**	**$\chi 2$**	**p**	**Z**
**ES vs. SS**	0.057	5.5	<1.0E-04	4.1
**ES vs. WS**	2.3E-06	24.5	<1.0E-04	8.1
**SS vs. WS**	1.3E-03	12.4	<1.0E-04	4.9
